# Renal pseudotumor mimicking renal cell carcinoma in an elderly patient with ovarian carcinoma: Case report and literature review

**DOI:** 10.1016/j.radcr.2024.11.063

**Published:** 2024-12-19

**Authors:** Maximilian Althammer, Thomas Kroencke, Josua A. Decker

**Affiliations:** Department of Diagnostic and Interventional Radiology**,** University Hospital Augsburg**,** Augsburg**,** Germany

**Keywords:** Renal pseudotumor, Ovarian carcinoma, Chronic interstitial nephritis, CT-guided biopsy, Nephrectomy, Overtreatment

## Abstract

This case report is about an 84-year-old female patient with a history of high-grade serous ovarian carcinoma who was diagnosed with a renal pseudotumor. Initial imaging in February 2023 showed signs of a renal cell carcinoma and possible lung metastases. A CT-guided biopsy and histopathological analysis ruled out malignancy and confirmed a benign inflammatory pseudotumor. This case underlines the importance of a biopsy to avoid unnecessary treatments such as nephrectomy and emphasizes the worth of comprehensive diagnostic evaluation. These steps are important to prevent unnecessary surgical interventions, overtreatment and to focus on effective, targeted therapy for the actual underlying pathology.

## Introduction

Renal pseudotumors are very rare, benign lesions that can mimic malignant tumors both clinically and radiologically which leads to a substantial diagnostic challenge. Their rarity and the high risk of a misdiagnosis highlight the challenge of distinguishing these lesions from malignant renal tumors to prevent unnecessary and potentially harmful interventions. The accurate identification of renal pseudotumors relies heavily on imaging techniques and histopathological confirmation underlining the critical need for accurate differential diagnosis. This case report aims to emphasize the diagnostic difficulties associated with renal pseudotumors and the necessary role of multidisciplinary teamwork in their identification and diagnosis. The necessity to publish is shown by this case report and demonstrates how a thorough diagnostic approach, including advanced imaging techniques, CT-guided biopsy and histopathological analysis helped solve a complex clinical scenario. Given the rarity of this pathology and its ability to feign malignancies, this report highlights the significance of raising awareness about renal pseudotumors in the medical community

## Case presentation

**Fig. 1:**
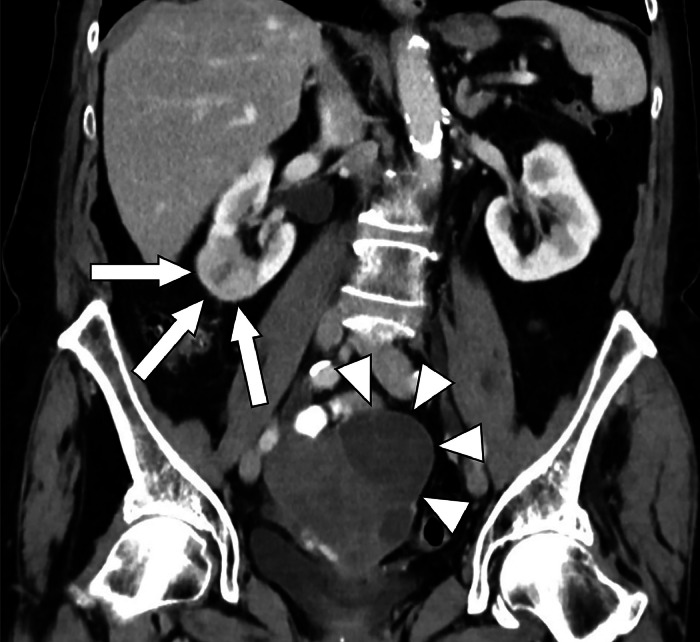
Initial imaging on February 10, 2023, by an external radiologist. Contrast-enhanced abdominal CT in venous phase, coronal reformation. This CT is showing a suspicious mass at the lower pole of the right kidney with central hypodensity (white arrows) and an inhomogeneously enlarged uterus with cystoid changes (white arrowheads).

The patient is an 84-year-old lady presenting arterial hypertension, atrial fibrillation, mitral valve insufficiency, hyperlipidemia, and earlier gastrointestinal bleeding under anticoagulation. A first imaging diagnosis was made on February 10, 2023, by an external radiologist. A CT-scan of the abdomen evidenced a 39 × 20 mm lesion at the lower pole of the right kidney (Fig. 1). The lesion was noted to have heterogeneous contrast enhancement with a hypoattenuating center, and the enhancement was lower than the renal cortex in the portal venous phase, raising suspicion for a malignant mass. Besides, there was a heterogeneous enlargement of the uterus with associated cystic changes. In the same CT, multiple pulmonary nodules were detected, predominantly in the left lower lobe and the right upper lobe, with the largest nodule measuring up to 9 mm. These imaging characteristics suggested possible metastases, leading to the suspected diagnosis of renal cell carcinoma with pulmonary metastases.

The case was discussed in an interdisciplinary tumor board and based on the suspicion of renal cell carcinoma with possible pulmonary metastases, a decision was made to proceed with a percutaneous biopsy of the mass of the right kidney.

**Fig. 2:**
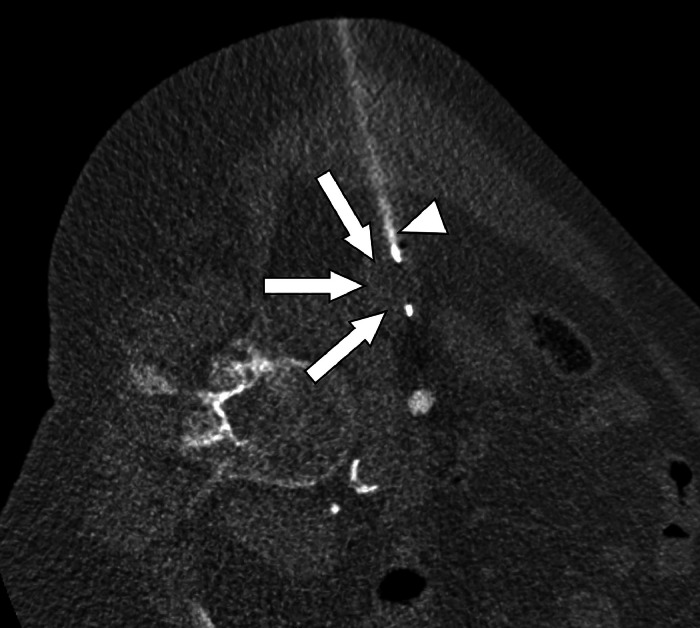
CT-guided biopsy with biopsy needle (white arrowhead) placed in the suspected mass on the lower pole of the right kidney (white arrows), on March 16, 2023. Unenhanced CT in left lateral decubitus position.

Further diagnostic investigations were thus proposed in the light of this discovery and were performed at the University Hospital of Augsburg. The patient refused further biopsy of the lung nodules, and no additional workup of those was performed in our hospital. However, outpatient CT-follow-up was planned to monitor the pulmonary nodules. CT-guided biopsy of the right kidney was performed on 16th March 2023 (Fig. 2). The biopsy procedure was conducted using an 18 G, 16 cm disposable semi-automatic biopsy needle (BARD Mission) with coaxial technique. The patient was placed in a left lateral decubitus position, and a lateral percutaneous approach was chosen. The biopsy was performed under sterile conditions with local anesthesia administered (20 mL of 1% Lidocaine). Histopathology revealed chronic interstitial nephritis with present features of inflammatory activity, lymphocytic infiltration, and spindle cell proliferation; no sign of malignancy. On microscopic inspection, there were predominantly tubules showing mild retention of secretion, without any area of neoplastic tissue. The immunohistochemical staining also showed B cells arranged as follicles, scattered T cells in the tissue, and a few IgG4+ plasma cells. Congo red staining did not reveal amyloid deposits.

Other ancillary studies in immunohistochemistry included congo red, CD20, CD3, and IgG4 staining, all performed on March 29th and 31st, 2023, which again supported the benign nature of the renal lesion and excluded amyloidosis or any feature of malignancy. Chronic interstitial nephritis, lymphocytic infiltration, no evidence of neoplastic cells—final pathology.

The basic laboratory data only disclosed increased paraproteinemia with Ig kappa light chains, without Bence-Jones proteinuria or any other criterion for multiple myeloma, such as hypercalcemia, renal failure, anemia, or bone lesions. An M-gradient was identified by an outside oncologist; thus, suspicion for multiple myeloma arose but it was classified as monoclonal gammopathy of undetermined significance.

The head MRI, performed on March 16, 2023, showed a small-sized ischemic lesion of the right parietal subcortex and a very mild postischemic remodeling of the left frontal region. No brain metastasis and/or meningeal carcinomatosis were pointed out. In the occipital lobe, it was noticed that there was the presence of a nonaggressive intraosseous lesion—unmodified compared with previous studies. This was assessed as arachnoid granulation. Imaging follow-up performed on July 6, 2023, showed bipulmonary nodules still present, with a worrisome consolidation in the left upper lobe. However, there was no significant growth in the nodules, and the follow-up confirmed their indeterminate nature without immediate intervention. Besides that, CT of the abdomen performed on July 11, 2023, demonstrated resolution of the previously identified renal lesion along with the detection of 2 new hypodense lesions in the mid-segment of the right kidney. Furthermore, the uterus was still enlarged and suspicious for malignancy; thus, follow-up was indicated.

An extended diagnostic workup has been carried out in view of the initial suspicion of renal cell carcinoma up until histopathology finally confirmed that the tumor of the kidney is, in fact, a benign inflammatory pseudotumor. Management thus addresses the now-established diagnosis of high-grade serous ovarian carcinoma in the patient.

On August 28, 2023, the following surgery was performed on this patient: bilateral adnexectomy, extended anterior rectal dissection, extended peritoneal dissection, omentectomy, and ureter resections followed by re-implantation in the bladder as part of treatment for ovarian cancer.

Histologically, the reports revealed high-grade serous ovarian carcinoma, described as pT2b, pN1b, FIGO IIIA 1ii, with no gross residual.

**Fig. 3:**
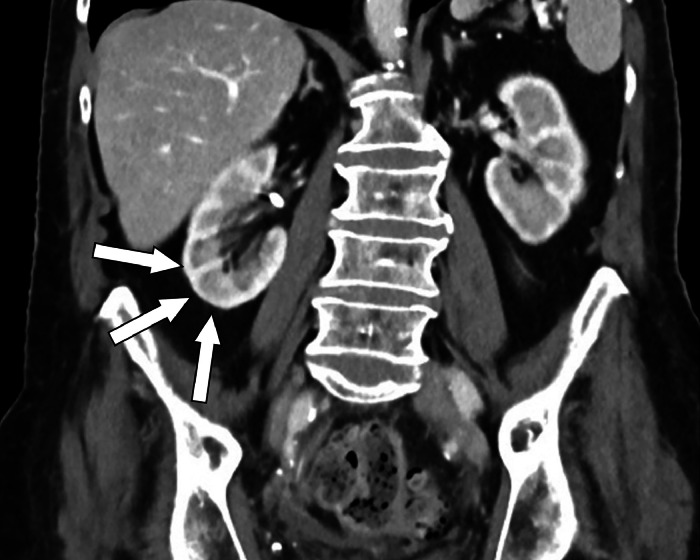
Follow-up imaging with contrast-enhanced abdominal CT on September 13th 2023. Contrast-enhanced abdominal CT in venous phase, coronal reformation. This CT showed no sign of renal mass on the right kidney (white arrows).

**Fig. 4:**
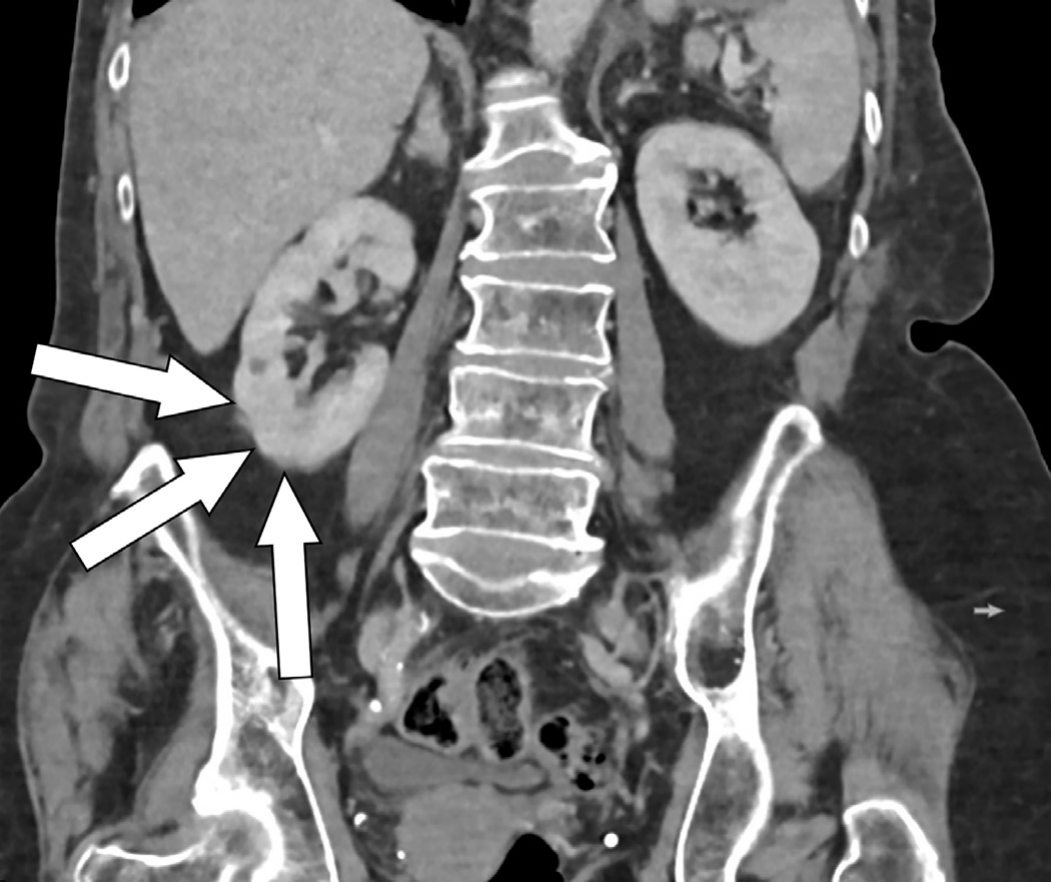
Follow-up CT on April 9, 2024 showing no sign of evidence of a tumor in the right kidney (white arrows). Contrast-enhanced abdominal CT in venous phase, coronal reformation.

Follow-up studies done on September 13, 2023, did not show any renal masses or significant abnormalities (Fig. 3). However, there was a small hematoma around the sacrum, degenerative changes in the spine, and an atrophic splenic infarction. Follow-up sonographic examination was also done in November 2023 to confirm the physiological status of the kidneys, showing no evidence of dilatation or mass for both the right and left kidneys. A renal scintigraphy carried out in December 2023 with 87 MBq of 99mTc-MAG3 showed no defects of the renal parenchyma and thus also a normal physiological renal clearance. Both kidneys were symmetrically supplied, considering both perfusion and excretion parameters, contributing 69% for the left and 31% for the right kidney to the total tubular function. The total tubular clearance was 181 mL/min, at the lower limit of normal values, without any anomalies indicative of prior interstitial nephritis or pseudotumor. Accordingly, an abdominal CT taken from an external clinic in April 2024 showed no evidence of either recurrence of a renal pseudotumor or malignant tumor (Fig. 4).

### Clinical course and management

The previous suspected diagnosis of renal cell carcinoma prompted a very thorough diagnostic approach. Histopathology played a essential role in preventing unnecessary nephrectomy by identifying the renal mass as a benign inflammatory pseudotumor rather than malignancy. A CT-guided biopsy was used as a less invasive diagnostic tool, which was particularly valuable in this complex case and helped to secure an accurate diagnosis. The biopsy and subsequent histopathologic analysis revealed chronic interstitial nephritis with lymphocytic infiltration and no neoplastic cells, so no aggressive kidney surgery was required.

Following confirmation of a benign renal pseudotumor, the clinical focus shifted from possible nephrectomy to treatment of the patient's advanced high-grade serous ovarian carcinoma. This change in management ensured that the patient received the best possible care, prioritizing her confirmed high-grade ovarian cancer over the benign renal findings.

Prognostically, the benign nature of the renal pseudotumor indicates a favorable future outlook for the kidney. However, the overall prognosis for the patient remains complex due to the advanced stage of her ovarian cancer. Following surgery for ovarian cancer on August 28, 2023, during which extensive procedures were performed, the absence of any residual macroscopic tumors provides a cautiously optimistic outlook, although continued surveillance and management will be essential.

## Discussion

Renal pseudotumors are very rare, benign lesions, which can clinically and radiologically resemble malignant tumors, posing a significant diagnostic challenge. These kinds of lesions are often found by chance and must be distinguished from malignant masses to prevent unnecessary treatments.

The initial presentation of our patient with conjectured renal mass and pulmonary lesions raised the strong hypothesis of metastatic renal cell carcinoma. The increased paraproteinemia led us to consider multiple myeloma, which was later classified as monoclonal gammopathy of undetermined significance (MGUS) due to the lack of CRAB criteria and Bence-Jones proteinuria.

The histopathological examination of the biopsied renal mass ultimately confirmed a benign inflammatory process, which significantly altered the clinical management of the patient and prevented unnecessary surgical actions. The final diagnosis of the renal pseudotumor was made more challenging due to the simultaneous presence of high-grade serous ovarian carcinoma. The ovarian cancer required extensive surgical treatment, including bilateral adnexectomy and rectal resection, resulting in no macroscopic tumor residue.

This discussion compares our results with those in the literature and highlights similarities and differences in diagnostic approaches, treatment strategies, and outcomes. In our case, the initial diagnosis was suspected renal cell carcinoma based on imaging studies, including CT scans that revealed a suspicious lesion. The final diagnosis was made by CT-guided biopsy, which identified chronic interstitial nephritis, a benign inflammatory process.

The case by Dell'Aprovito N et al. [1] shows similarities to our case, in which a 73-year-old woman was diagnosed with xanthogranulomatous pyelonephritis, resembling renal cell carcinoma, based on ultrasound, CT, and ultimately nephrectomy with histology. Cai YI et al. [2] reports on an 80-year-old woman with IgG4-related inflammatory pseudotumor, also diagnosed through comprehensive imaging, including ultrasound, CT, PET-CT, and eventually histological analysis after nephrectomy.

Other studies and case reports [[3], [4], [5], [6]] with diagnoses such as chronic glomerulonephritis, inflammatory pseudotumor, and renal splenosis relied on combinations of imaging procedures and ultimately nephrectomy. These cases underscore the importance of advanced imaging techniques for initial diagnosis. However, our case demonstrates that biopsy can provide a definitive diagnosis without the need for nephrectomy, suggesting a potentially less invasive approach.

Instead of performing direct nephrectomy, as in other studies, our case opted for conservative treatment based on the results of the CT-guided biopsy, which confirmed the benign nature of the renal pseudotumor. This differs from the case in Dell'Aprovito N et al. [1], where nephrectomy was performed due to suspicion of malignancy. Similarly, in the study by Cai YI et al. [2], a nephrectomy was conducted after extensive imaging. The other compared studies [[3], [4], [5], [6]] also chose direct nephrectomy as the primary treatment based on the clinical presentation and imaging of the patients.

The main difference between our case and the other cases compared in this report is our decision to opt for a less-invasive approach to the suspected renal lesion. We avoided unnecessary nephrectomy, which we believe is advantageous in cases where a benign diagnosis can be confirmed by less invasive means. This approach can reduce the risk of surgical complications and ultimately preserve kidney function. In our case, pathology confirmed the benign nature of the mass. Similarly, in the case reports by Dell'Aprovito N et al. [1] and Cai YI et al. [2], histopathological exploration after nephrectomy also verified benign lesions, including inflammatory pseudotumors.

Reports and studies [[3], [4], [5], [6]] diagnosed different benign processes in renal masses after nephrectomy, highlighting that benign lesions may be identified with appropriate diagnostic tools. Our case, along with these reports, supports the notion that less invasive approaches, such as biopsy and in-depth histopathological assessment, may suffice, thus avoiding nephrectomy.

In addition to biopsy, imaging techniques such as contrast-enhanced ultrasound (CEUS), CT, and MRI play a significant role in differentiating between benign and malignant renal lesions. Recent studies suggest that CEUS, which utilizes second-generation contrast agents, may be particularly useful in distinguishing renal pseudotumors from true neoplasms. Mazziotti et al. [7] demonstrated that CEUS can provide comparable diagnostic accuracy to multiphase CT and dynamic MRI, with the advantage of being less invasive and more cost-effective. In their study of 24 patients, CEUS was used when conventional ultrasound and Doppler results were inconclusive. The findings showed equal perfusion and reperfusion in both the pseudotumor and surrounding parenchyma during the early and late corticomedullary phases, allowing for accurate differentiation from malignant tumors.

CT remains the standard imaging technique in many cases due to its widespread availability and ability to assess the size, location, and density of renal masses. In our case, CT revealed the initial lesion in the right kidney, which prompted further investigation. However, MRI offers superior soft tissue contrast and may be more sensitive in characterizing complex cystic lesions and distinguishing between benign and malignant masses. For example, MRI was important in ruling out brain metastases in this patient. Furthermore, PET-CT, as used in Cai YI et al.'s case [2], provides functional imaging that can assess metabolic activity within renal lesions, although it is less commonly used in diagnosing renal pseudotumors.

Given the diagnostic overlap between benign pseudotumors and malignant renal neoplasms, CEUS offers an attractive alternative to more invasive imaging modalities. As seen in Mazziotti et al.'s study [7], CEUS could reliably distinguish pseudotumors without the need for biopsy in some cases. This imaging modality could serve as a first-line diagnostic tool, particularly in patients for whom exposure to radiation or contrast agents (as used in CT or MRI) is contraindicated. Integrating CEUS into the diagnostic pathway for renal masses could help reduce unnecessary nephrectomies, improve patient outcomes, and minimize healthcare costs.

This case reinforces the need for a comprehensive diagnostic approach to differentiate malignant renal masses from benign pseudotumors. Using biopsy and histopathological examination, rather than proceeding directly to nephrectomy, offers a less invasive and effective diagnostic alternative, as seen in our case, where the risk of unnecessary surgical intervention was minimized, and renal function was preserved (Table 1).

**Table 1:** comparison of different variables of the investigated cases.

Study/Case report	Diagnosis	Age and sex	Diagnosing techniques	Symptoms	Treatment
Our own case	High grade serous ovarial carcinoma + benign inflammatory process (inflammatory renal pseudotumor)	84, w	Ultrasound, Abdominal CT, CT-Guided Biopsy, Renal scintigraphy	asymptomatic	Watch and wait, excision of the ovarian ca + postoperative treatment
Xanthogranulomatous pyelonephritis mimicking a renal cell carcinoma: a unique and challenging case. [1]	Xanthogranulomatous pyelonephritis mimicking renal cell carcinoma	73, w	Ultrasound, CT, MRI, radical nephrectomy: Histopathology	asymptomatic	Radical nephrectomy
IgG4-related inflammatory pseudotumor of the kidney mimicking renal cell carcinoma: A case report. [2]	IgG4-related inflammatory renal pseudotumor	80, w	Ultrasound, CT, PET-CT, Nephrectomy with subsequent histopathological analysis	asymptomatic	Nephrectomy, IgG4-RD with corticosteroids, no additional therapy was needed postsurgery
Rare renal pseudotumor associated with chronic glomerulonephritis mimicking renal cell carcinoma. [3]	Chronic glomerulonephritis associated with renal pseudotumor	17, m	CT, MRI, nephrectomy and following histology	abdominal pain, diarrhea, anuria, systemic edema	Nephrectomy
Inflammatory pseudotumor of the kidney: A case report. [4]	Inflammatory pseudotumor	10, w	Ultrasound, CT, nephrectomy with Histopathology	intermittent fever and headache	Nephrectomy
Inflammatory pseudotumor of the kidney: a case report. [5]	Renal inflammatory pseudotumor	57, m	Contrast-enhanced CT, ultrasound, radical nephrectomy	gross hematuria, left lumbar pain	Nephrectomy
Renal pseudo-tumor related to renal splenosis: Imaging features. [6]	Renal splenosis	29, ?	Ultrasound, CT, MRI, partial nephrectomy, histopathology	dysuria	Nephrectomy

## Conclusion

In challenging scenarios, our case demonstrates how performing a minimally invasive percutaneous biopsy coupled with histopathological analysis plays a very important role in accurately distinguishing and diagnosing renal tumors. The detailed diagnostic approach, which involved using a CT-guided biopsy, successfully identified an inflammatory pseudotumor, thus avoiding unnecessary surgical interventions. The diagnostic process began with imaging that initially indicated the presence of cancer, followed by an analysis that considered conditions such as renal cell carcinoma and multiple myeloma. The decisive turning point was the CT-guided biopsy, which dispelled concerns about cancer and confirmed the benign nature of the tumor. This allowed us to shift focus to the treatment of the underlying high-grade ovarian carcinoma. The follow-up imaging in September 2023 and April 2024 confirmed the benignity of the pseudotumor and underpinned the effectiveness of our diagnostic strategy.

This case emphasizes the importance of a multidisciplinary approach, where radiologists, oncologists, and pathologists worked collaboratively to avoid unnecessary surgery. Such collaboration ensured accurate diagnosis and prevented overtreatment.

Future cases should prioritize less invasive diagnostic techniques, such as biopsy, before considering nephrectomy in radiologically suspicious lesions. This approach minimizes surgical risks, especially in elderly patients with multiple comorbidities.

In addition, CEUS can be considered as a useful imaging modality alongside traditional techniques to further aid in the differentiation of benign and malignant renal lesions.

Future research should aim at the evaluation of a recommendation for action on the diagnosis and treatment of renal pseudotumors and advocate for less invasive histopathological confirmation of the final diagnosis of renal pseudotumors. By integrating biopsy and advanced imaging techniques like CEUS, unnecessary surgical interventions can be avoided, improving outcomes for patients and preserving kidney function.

## Patient consent

Renal pseudotumor in an 84-year-old female patient with ovarian carcinoma: case report and review of the literature: Written, informed consent for publication of her case and especially her CT images was obtained from the patient.
